# Legalized Cannabis in Colorado Emergency Departments: A Cautionary Review of Negative Health and Safety Effects

**DOI:** 10.5811/westjem.2019.4.39935

**Published:** 2019-06-03

**Authors:** Brad A. Roberts

**Affiliations:** University of New Mexico, Department of Emergency Medicine, Albuquerque, New Mexico Partner, Southern Colorado Emergency Medicine Associates, Pueblo, Colorado

## Abstract

Cannabis legalization has led to significant health consequences, particularly to patients in emergency departments and hospitals in Colorado. The most concerning include psychosis, suicide, and other substance abuse. Deleterious effects on the brain include decrements in complex decision-making, which may not be reversible with abstinence. Increases in fatal motor vehicle collisions, adverse effects on cardiovascular and pulmonary systems, inadvertent pediatric exposures, cannabis contaminants exposing users to infectious agents, heavy metals, and pesticides, and hash-oil burn injuries in preparation of drug concentrates have been documented. Cannabis dispensary workers (“budtenders”) without medical training are giving medical advice that may be harmful to patients. Cannabis research may offer novel treatment of seizures, spasticity from multiple sclerosis, nausea and vomiting from chemotherapy, chronic pain, improvements in cardiovascular outcomes, and sleep disorders. Progress has been slow due to absent standards for chemical composition of cannabis products and limitations on research imposed by federal classification of cannabis as illegal. Given these factors and the Colorado experience, other states should carefully evaluate whether and how to decriminalize or legalize non-medical cannabis use.

## INTRODUCTION

As of January 2018 in the United States, nine states have legalized cannabis for recreational use, with another 29 legalizing it for medical use. These policy changes have created broad interest in understanding the effects on public health and the healthcare system.

The Colorado Department of Public Safety report, “*Impacts of Marijuana Legalization in Colorado: A Report Pursuant to Senate Bill 13-283,”* includes a timeline for marijuana legalization in Colorado with five distinct periods in both the legal status and commercial availability of marijuana in Colorado.^1^ These include the following:

- Prior to 2000: It is illegal to possess or grow marijuana.
- 2000–2009: Amendment 20 is approved and medical marijuana is legalized. The Colorado Department of Public Health and Environment (CDPHE) issues registry identification cards to individuals who have received recommendations from a doctor that marijuana will help a debilitating medical condition. No regulated market exists. Individual grow operations or caregiver grow operations limited to five patients are allowed.
- 2010–2012: Medical marijuana is commercialized and regulated with licensed dispensaries, grow operations, and product manufacturers open in jurisdictions allowing these types of businesses. This corresponded with the Ogden memorandum issued in October 2009, which instructed U.S. Attorneys not to “focus federal resources in your States on individuals whose actions are in clear and unambiguous compliance with existing state laws providing for the medical use of marijuana.”^2^ The commercialization of medical marijuana followed and the number of patients registered with CDPHE increased dramatically from about 5000 in 2009 to almost 119,000 in 2011.
- 2013: Amendment 64 takes effect. Personal possession and grow limits for recreational marijuana are in place but sales are not commercialized. Medical continues as a regulated, commercial market.
- 2014 to present: Recreational and medical marijuana is fully regulated and commercialized. Licensed retail stores open January 1, 2014. This corresponded with the Cole memorandum, which gave further guidance to U.S. Attorneys: “[I]n jurisdictions that have enacted laws legalizing marijuana in some form and that have also implemented strong and effective regulatory and enforcement systems to control the cultivation, distribution, sale, and possession of marijuana, conduct in compliance with those laws and regulations is less likely to threaten federal priorities… [E]nforcement of state law by state and local law enforcement and regulatory bodies should remain the primary means of addressing marijuana-related activity.”^3^ This memorandum was widely interpreted to mean that the federal government would not interfere with state marijuana laws;^4^ however, in January 2018 the Cole memorandum was rescinded by then U.S. Attorney General Jeff Sessions.^4^

**Figure 1:**
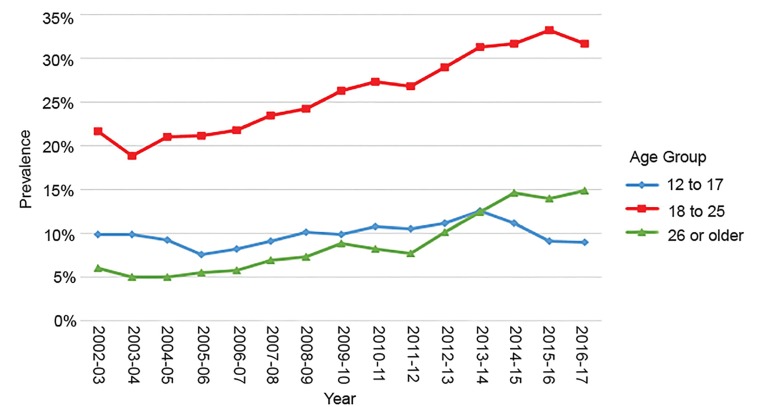
Marijuana use in the past month in Colorado, by age group. Reproduced from Substance Abuse and Mental Health Services Administration National Survey on Drug Use and Health: State Estimates. Available at: https://pdas.samhsa.gov/saes/state. Accessed November 2018.

**Figure 2:**
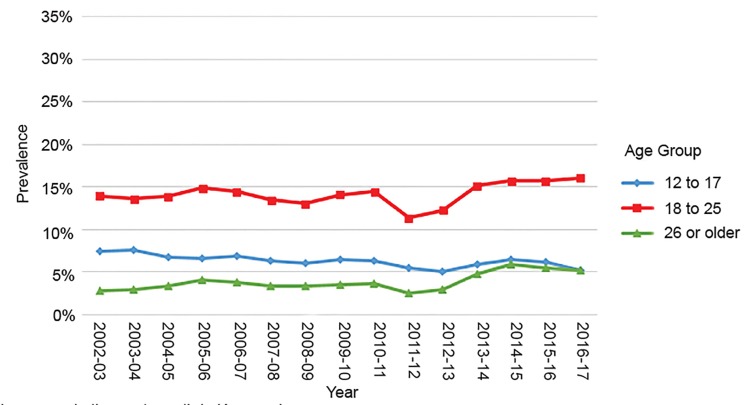
Marijuana use in the past month in Kansas, by age group. Reproduced from Substance Abuse and Mental Health Services Administration National Survey on Drug Use and Health: State Estimates. Available at: https://pdas.samhsa.gov/saes/state. Accessed November 2018.

Changes in past-month cannabis use by year and age group for Colorado and Kansas (non-legalized state) are shown in Figures 1 and 2. (Kansas was chosen for proximity; other non-legalized states in proximity, including Wyoming and Idaho, had similar graphs to Kansas.)^5^

Over this time span cannabis potency has increased. Current commercialized cannabis is near 20% tetrahydrocannabinol (THC), the primary psychoactive constituent of cannabis, while in the 1980s concentration was <2%. This 10-fold increase in potency does not include other formulations such as oils, waxes, and dabs, which can reach 80–90% THC.^6^

This general increase in cannabis use and increase in cannabis potency has led to cannabis-related presentations to emergency departments (ED) and hospitalizations across the state. This review will focus on negative health and safety effects Colorado has experienced with inclusion of relevant peer-reviewed literature. It will conclude with a short review of the medicinal use of cannabis products.

**Population Health Research Capsule**

What do we already know about this issue?

Legalized cannabis has led to increased cannabis related presentations to emergency departments (ED).

What was the focus of this review?

The negative impacts to EDs, particularly in the state of Colorado, following cannabis legalization.

What was the conclusion of this review?

Cannabis legalization has been correlated with multiple adverse outcomes that impact EDs.

How does this improve population health?

Healthcare policy makers may take the adverse outcomes described into consideration when considering if and/or how to legalize cannabis.

### Cannabis Effects on Healthcare Resources in Colorado

ED visits and hospitalizations with marijuana-related billing codes have increased following legalization. Mental illness represents a concerningly large number of marijuana-related visits. A retrospective review by Wang et al. reported Colorado Hospital Association hospitalizations and ED visits with marijuana-related billing codes. Between 2000 and 2015, hospitalization rates increased 116% from 274 to 593 per 100,000 hospitalizations. For primary diagnosis categories, the prevalence of mental illness was five-fold higher (5.07; 95% confidence interval [CI], 4.96 – 5.09) for ED visits and nine-fold higher (9.67; 95% CI, 9.59 – 9.74) for hospital admissions for patients with marijuana-related billing codes compared to those without.^7^ This data compared diagnostic categories between patients with a marijuana-related diagnostic code and those without.

**Figure 3:**
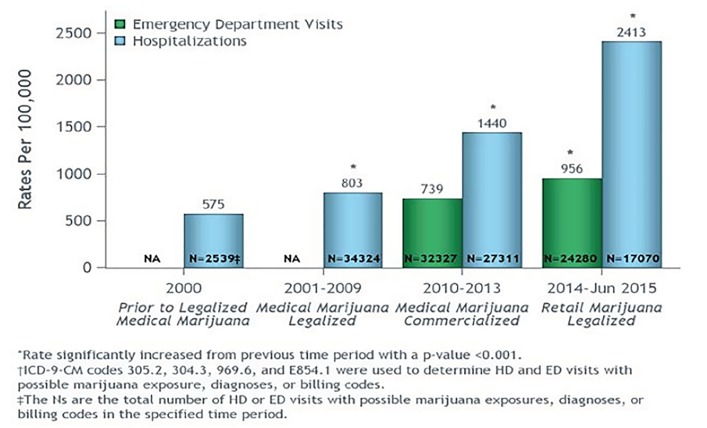
Rates of hospitalizations (HD) and emergency department (ED) visits per year with possible marijuana exposures, diagnoses, or billing codes per 100,000 HD and ED visits, by legalization eras in Colorado. *NA*, Data not available. Data provided by Colorado Hospital Association with analysis provided by Colorado Department of Public Health and Environment. Note: Data for 2015 covers January 1, 2015 – June 30, 2015. An individual can be represented more than once in the data; therefore, the rate is HD or ED visits with marijuana codes per 100,000 total HD or ED visits. Reproduced from Marijuana Legalization in Colorado: Early Findings. A Report Pursuant to Senate Bill 13-283. Colorado Department of Public Safety. 2016. Available at: http://cdpsdocs.state.co.us/ors/docs/reports/2016-SB13-283-Rpt.pdf. Accessed March 2018.

Subsequent data by the CDPHE show significant increases in hospitalizations in each phase of marijuana legalization, increasing from 575 per 100,000 hospitalizations in 2000 to 2413 in the 2014–June 2015 period, as displayed in Figure 3.^8^ There are differences in incidence between the Wang study and the CDPHE report because the Wang study only included a patient’s healthcare event if a marijuana code was among the first three diagnostic codes, while the CDPHE study included marijuana diagnostic codes within the top 30.

**Figure 4:**
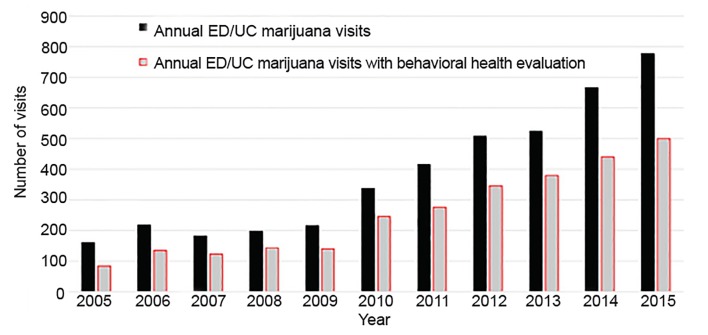
Number of emergency department (ED)/urgent care (UC) visits with cannabis-associated International Classification of Diseases codes or positive urine drug screens by adolescents aged 13 to < 21 to a tertiary-care children’s hospital system in Colorado by year.^105^

ED and urgent care (UC) visits with cannabis-associated International Classification of Diseases (ICD) codes or positive urine drug screens for teenagers and young adults have increased since legalization, and the majority require behavioral health evaluation. A subsequent retrospective review by Wang et al. from 2005–2015 identified 4202 such visits for patients 13 to <21 years old to a tertiary-care children’s hospital system. Behavioral health evaluation was obtained for 2813 (67%) and a psychiatric diagnosis was made for the majority (71%) of the visits. ED/UC visits with cannabis-associated ICD codes or positive urine drug screens of all types increased 2.7-fold from 1.8 per 1000 in 2009 to 4.9 per 1000 in 2015 (N = 161 in 2005 to 777 in 2015). Behavioral health consultations increased 2.7-fold from 1.2 per 1000 in 2009 to 3.2 per 1000 in 2015 (N = 84 in 2005 to 500 in 2015). These data indicate that despite national survey data suggesting the rate of adolescent marijuana use is flat, there has been a significant increase in adolescent ED/UC visits with cannabis-associated ICD codes or positive urine drug screens.^9^ Figure 4 displays these visits by year.

### Cannabis Effects on Mental Health

#### Psychosis and Schizophrenia

Previous studies, including large reviews by the World Health Organization (WHO) and the National Academies of Sciences, Engineering, and Medicine (NASEM), have found substantial evidence of a statistical association between cannabis use and the development of schizophrenia or other psychoses, with the highest risk among the most frequent users.^6^,^10^ In a study of 45,570 Swedish men drafted into the military, the authors found that the men who had tried cannabis by age 18 were 2.4 times (95% CI, 1.8–3.3) more likely to be diagnosed with schizophrenia over the next 15 years than those who had not.^11^ A follow-up study found a dose-response relationship between frequency of cannabis use at the age of 18 and the risk of schizophrenia. This effect persisted after controlling for confounding factors such as psychiatric diagnosis at enlistment, IQ score, personality variables concerned with interpersonal relationships, place of upbringing, paternal age, cigarette smoking, disturbed behaviors in childhood, history of alcohol misuse, family history of psychiatric illness, financial situation of the family, and father’s occupation. (The enlistment procedure included intelligence tests and non-anonymous, self-reported questionnaires on family, social background, behavior during adolescence, and substance use – including first drug used, drug most commonly used, frequency of use, and direct questions regarding use of a list of specified drugs.) The researchers estimated that 13% of cases of schizophrenia could have been averted if no one in the cohort had used cannabis.^12^ These findings have been reproduced repeatedly and across the world.^13^–^20^

#### Depression, Anxiety, and Suicide

Cannabis use is associated with increased rates of depression, anxiety, and suicide. The NASEM found that there is a moderate statistical association between cannabis use and an increased risk for the development of depressive disorders (odds ratio [OR] = 1.17; 95% CI, 1.05–1.30) and this increases with increased frequency of use (OR = 1.62; 95% CI, 1.21–2.16).^10^,^21^ There was also moderate evidence of a statistical association between regular cannabis use and increased incidence of social anxiety disorder (OR = 1.28; 95% CI, 1.06–1.54).^10^,^22^ The NAESM found that there was moderate evidence of a statistical association between cannabis use and the incidence of suicidal ideation (OR = 1.43; 95% CI, 1.13–1.83 with any cannabis use, OR = 2.53; 95% CI, 1.00–6.39 with heavy cannabis use) and suicide attempts (OR = 2.23; 95% CI, 1.24–4.00 for any cannabis use, OR = 3.20; 95% CI, 1.72–5.94 with heavy cannabis use), and increased incidence of suicide completion (OR = 2.56; 95% CI, 1.25–5.27 for any cannabis use).^10^,^23^

The NASEM reviewed multiple studies to come to the summary conclusions, and the odds ratios represent the most compelling systematic review for the conclusions. However, there were many more studies used to reach the stated conclusions. The data reviewed by the World Health Organization also demonstrate similar results for depression, anxiety, and suicide.^6^ Both the NASEM and the WHO reviews acknowledge that reverse causation and shared risk factors cannot be ruled out as explanations of these statistical associations and acknowledge that further research is needed.

**Figure 5:**
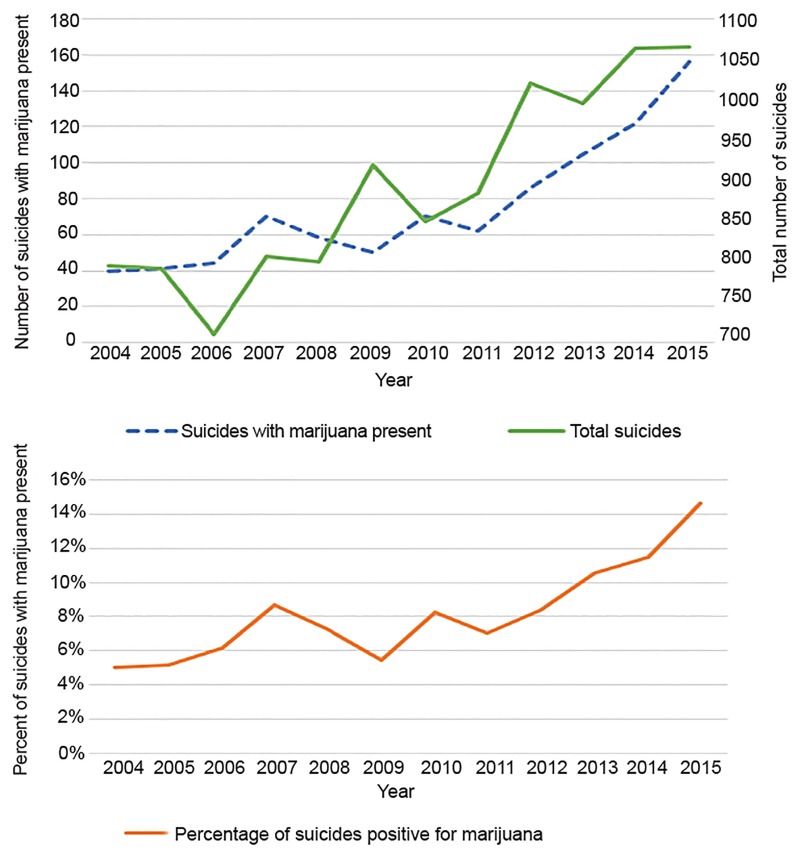
Suicides with marijuana toxicology by year and total suicides by year in Colorado (A). Percent of suicides with marijuana present by year (B).^24^

In the most recent data on Colorado adolescent suicides, marijuana was the most common substance present for ages 10–19 in 2016. Of 62 suicides with toxicology data available, marijuana was present in 30.6% (n = 19) compared to 9.7% (n = 6) for alcohol.^24^ This trend has been increasing since liberalization of marijuana policy in 2010. This is more concerning as suicide is currently the leading cause of death of adolescents in Colorado.^25^ For all age groups in Colorado, in the five-year period from 2004–2009 there were 4822 suicides and 7.1% (n = 303) of those were marijuana positive on toxicology analysis (538 did not have toxicology data available). In the subsequent five-year period of marijuana legalization, 2010–2015, there were 5880 total suicides (22% increase), and 12.6% had a positive toxicology for marijuana (n = 601; 1,120 did not have toxicology data available). This represents a statistically significant 77.5% increase in the proportion of suicide victims with toxicology positive for marijuana (an absolute difference of 5.5%) for which toxicology data were reported (chi square 77.2884, p<0.0001). Suicides with marijuana toxicology by year and overall suicide by year data are displayed in Figure 5.

#### Social Outcomes

Cannabis has been associated with adverse social outcomes which may impact EDs and patient health. The large (N = 49,321) cohort study of Swedish men drafted at age 18–20 and followed to age 40 showed increased risk of unemployment and need for welfare assistance in those using cannabis greater than 50 times (risk ratio [RR] = 1.26; 95% CI, 1.04–1.53 for unemployment), (RR = 1.38; 95% CI, 1.19–1.62 for welfare assistance).^26^ These results were repeated in a longitudinal birth cohort study in New Zealand to 25 years old, which found high levels of cannabis use correlated with statistical significance to poorer educational outcomes, lower income, greater welfare dependence and unemployment, and lower relationship and life satisfaction. This cohort was classified into six levels of cannabis use, and found that as cannabis use increased, the odds ratio of adverse outcome increased.^27^,^28^ Both of these studies adjusted for confounding factors including socioeconomic background of the family, family functioning and exposure to adversity, exposure to child sexual and physical abuse, childhood and adolescent adjustment, academic achievement in early adolescence, comorbid mental health disorders, and other substance use.

A prospective cohort study from upstate New York (N = 548) found that, compared with cannabis nonusers or minimal users (a few times a year or less), chronic users and users who began use in early adulthood and then tapered off use into later adulthood, had a significantly higher likelihood of unemployment at mean age 43 (adjusted OR = 3.51; 95% CI, 1.13–10.91), even after controlling for covariates.^29^ The NASEM review stated that there was a limited level of evidence of impaired academic achievement and education outcomes, increased rates of unemployment and/or low income, and impaired social functioning or engagement in developmentally appropriate social roles.^10^ The report stated that although there was evidence to suggest these outcomes, it was difficult to document a direct link between cannabis use and these outcomes because other variables played a role. Social outcome data for cannabis users specifically in Colorado are currently unavailable and could be an area for further research.

#### Structural, Functional, and Chemical Brain Changes in Cannabis Users

A number of review articles on cannabis have described adverse effects on brain imaging.^6^,^30^–^33^ These findings may help establish a mechanistic link between the epidemiological studies on the adverse effects of cannabis. Structural, functional, and chemical changes to the brain have been established. These include both the gray matter (neuronal cells) and white matter (nerve axons responsible for communication).^34^,^35^ Structural changes to the brain include reductions in the hippocampus^34^–^38^ (12.1% in the left and 11.9% in the right, relative to controls)^38^ and amygdala^37^,^38^ (6.0% in the left and 8.2% in the right, relative to controls)^38^ volumes in cannabis users.

Several studies also identified reductions in volume of specific areas of the prefrontal cortex,^39^–^41^ as well as functional magnetic resonance imaging (fMRI) studies demonstrating reduced functional connectivity in the prefrontal networks responsible for executive function (including inhibitory control) and the subcortical networks, which process habits and routines.^30^,^42^–^25^ Other fMRI studies show reduced connectivity in the fimbriae of the hippocampus and commissural fibers extending to the precuneus, and suggest that this disturbed brain connectivity in cannabis users may underlie cognitive impairment and vulnerability to psychosis, depression, and anxiety disorders.^46^ Multiple other areas of the brain have also been shown to demonstrate changes on fMRI studies in response to cannabis and include the orbitofrontal cortex, anterior cingulate cortex, striatum, amygdala, hippocampus, and cerebellum.^37^ In general, these changes on both structural and functional MRI studies corresponded with frequency of use and earlier age of onset of use (although several studies identified these changes in adult users as well).^34^,^35^

Changes to neurotransmitters in the brain have also been well described in systematic reviews and include disruptions in glutamate,^47^ dopamine,^48^ N-acetylaspartate,^49^ myo-inositol,^49^ choline,^49^ and γ-aminobutyric acid (GABA).^33^,^49^

Taken together, these changes may underlie the clinical features being observed in observational and epidemiological studies demonstrating increases in psychosis, impulsivity, depression, anxiety, suicidality, decreases in cognition, IQ, and executive function, abnormalities in habits, routines, decision-making capacity, and deficits in learning, memory, attention, and social interaction.^6^,^30^,^31^

#### Link to Other Substance Abuse

**Figure 6:**
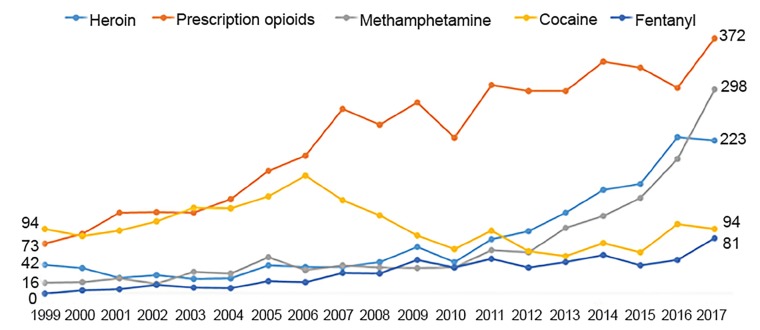
Drug poisoning/overdose deaths in Colorado by involvement of specific drug type: Colorado residents, 1999–2017.* Reproduced from: Vital Statistics Program, Colorado Department of Public Health and Environment. Available at: https://drive.google.com/file/d/1vfi4kL9eD9rib7aEboteiGw67gOxXFpf/view *Drug categories are not mutually exclusive; a death involving more than one type of specific drug will be counted in each applicable category. “Fentanyl” is a subset of “prescription opioid.”

Cannabis use has also been associated with abuse of other illicit substances. According to the NASEM report, there is a moderate level of evidence of a statistical association between cannabis use and the development of substance dependence and/or substance abuse disorder for alcohol, tobacco, and illicit drugs.^10^ Multiple cohort studies have demonstrated these results.^50^–^52^ Four separate discordant twin studies have found that the twin who used marijuana was more likely to use other substances even after controlling for environmental and genetic influences.^53^–^56^ Although some studies reported that medical cannabis has resulted in improvements in opiate-related deaths,^57^,^58^ Colorado has had an increase in poisoning and deaths from opiates and methamphetamines since 2010, with the highest in 2017. These rates have increased nationwide as well and the influence of cannabis in Colorado is difficult to discern. Nevertheless, the increase in overdose deaths in Colorado is alarming. These data are shown in Figure 6.^25^

Although animal studies do not consistently translate to human effects, rat studies can provide some mechanistic clues. After exposure to tetrahydrocannabinol (THC), rats have an increased behavioral sensitization response to not only THC but also opiates and nicotine.^59^–^61^ Studies also demonstrate that these behavioral changes in rats correspond to neuronal activity changes in mesolimbic dopamine neurons in the ventral tegmental area and nucleus accumbens and that cross-tolerance results with exposure to morphine, amphetamines, and cocaine.^61^,^62^ Repeat morphine self-administration has been shown to be significantly lower in CB_1_ knockout mice (CB_1_ receptors are the among the most predominant G protein-coupled receptors in the brain and mediate most of the psychotropic effects of THC) and opiate withdrawal symptoms significantly less when the knockout mice are administered naloxone.^63^

#### Cannabis Dependence/Withdrawal Symptoms

Cannabis use may result in dependence and cessation may result in withdrawal symptoms. Dependence rates are reported at one in 10 among those who ever use cannabis, one in six among adolescent users, and one in three among daily users.^6^,^64^–^67^ Withdrawal symptoms may include anxiety, insomnia, appetite disturbance, and depression. These symptoms are sufficient to impair everyday functioning and are markedly attenuated by doses of an oral cannabis extract.^6^

### Other Relevant Physiological and Safety Concerns with Cannabis

#### Cannabinoid Hyperemesis Syndrome

Cannabinoid hyperemesis syndrome (CHS) has been well described in the literature.^68^–^70^ The symptoms of CHS include significant nausea, violent vomiting, and abdominal pain in the setting of chronic cannabis use. Cardinal diagnostic characteristics include regular cannabis use, cyclic nausea and vomiting, and compulsive hot baths or showers with resolution of symptoms after cessation of cannabis use.^69^ CHS patients present similarly to cyclic vomiting syndrome patients with the exception that cannabis use is required to make the diagnosis.^69^ Following legalization, the prevalence of cyclic vomiting presentations to Denver Health and the University of Colorado Hospital increased 1.92-fold (95% CI, 1.33 to 2.79) from 41 per 113,262 ED visits from a year prior to marijuana liberalization (November 1, 2008–October 31, 2009) to 87 per 125,095 ED visits a year following marijuana liberalization (June 1, 2010–May 31, 2011). Patients with cyclic vomiting in the post-liberalization period were more likely to have marijuana use documented than patients in the pre-liberalization period (OR = 3.59; 95% CI, 1.44 to 9.00).^71^ These patients often are evaluated with multiple imaging studies, lab work, endoscopies, and admissions to the hospital as well as antiemetic treatment. These studies are often non-diagnostic and treatment is often ineffective. This may also influence ED crowding.^68^,^72^

#### Motor Vehicle Collisions

Traffic fatalities with blood or urine drug screens positive for cannabinoids have sharply risen across Colorado.^73^,^74^ Total fatal motor vehicle collisions (MVC) in Colorado had been decreasing from a high of 677 in 2002 to a low of 407 in 2011 but then began increasing each year since then to 600 in 2017. Total MVCs mirror this trend. The NASEM review found substantial evidence of a statistical association between cannabis use and increased risk of MVCs.^10^ CDPHE found substantial evidence that recent marijuana use by a driver increases his or her risk of a MVC and that the higher the blood THC level, the higher the risk of MVC. The use of alcohol and marijuana together increases risk of impairment and MVC more than either substance alone. For less-than-weekly marijuana users, using marijuana containing 10 milligrams (mg) of THC is likely to impair the ability to safely drive, bike, or perform other safety-sensitive activities. A typical marijuana cigarette, or joint, contains 60–115 mg of THC.^75^

A systematic review of observational studies and meta-analysis for acute cannabis consumption and MVC risk found that driving under the influence of cannabis was associated with a significantly increased risk of MVCs compared with unimpaired driving (OR = 1.92; 95% CI, 1.35 to 2.73), especially for fatal collisions (OR = 2.10; 95% CI, 1.31 to 3.36).^76^ However, a recent study of crash fatality rates after recreational marijuana legalization in Washington and Colorado found changes in motor vehicle crash fatality rates were not statistically different from those in similar states without recreational marijuana legalization. This was, however, only after further statistical regression analysis (for population, gender, spending on road construction and maintenance, annual gross domestic product, per capita income, unemployment rate, per capita alcohol consumption, seatbelt laws, road density, traffic density, and rurality). Initial data demonstrated that after legalization, motor vehicle crash fatality rates increased by a mean of +0.1 (± 0.4) fatalities per billion vehicle miles traveled in Washington and Colorado, and decreased by a mean of −0.5 (± 0.9) fatalities per billion vehicle miles traveled in the control states each year.^77^

#### Cardiovascular Effects

The effect of cannabinoids on the cardiovascular system is complex and an area of ongoing research.^78^ Of concern to practicing emergency physicians is ST-segment elevation myocardial infarctions and acute stroke presentations with a close temporal relationship with cannabis use, which have been documented in multiple case reports in otherwise young, healthy, male patients.^79^–^82^ The NASEM summary found there was a limited level of evidence of a statistical association between acute cannabis use and triggering an acute myocardial infarction (AMI), ischemic stroke, or subarachnoid hemorrhage.^10^ The WHO review states: “There is evidence that cannabis use can trigger coronary events. Recent case reports and case series suggest that cannabis smoking may increase cardiovascular disease risk in younger cannabis smokers who are otherwise at relatively low risk.”^6^

CDPHE found moderate evidence that marijuana use increases risk of ischemic stroke in individuals younger than 55 years of age and limited evidence that acute marijuana use increases risk of myocardial infarction.^75^ The main case crossover study cited for the AMI findings demonstrated that the risk for AMI associated with cannabis use during the hour preceding symptoms of AMI was elevated 4.8 times over baseline (95% CI, 2.9–9.5). This risk was substantially reduced following that hour.^83^

A review of nationwide inpatient sample data from 2010 to 2014 demonstrated a 32% increase in inpatient admissions for primary diagnosis of myocardial infarction and secondary diagnosis of cannabis use disorder (increasing from 2198 to 2900 cases). The overall mean age of patients was 41 years old. These patients also had longer lengths of stay, higher hospitalization costs, and higher levels of morbidity due to AMI following hospitalization than non-cannabis users.^84^

In a study reviewing secondhand marijuana smoke exposure, the authors found that one minute of exposure substantially impaired endothelial function in rats for at least 90 minutes, considerably longer than comparable impairment by tobacco secondhand smoke.^85^

The pathophysiological basis of these events is not fully understood and a full discussion is beyond the scope of this review. In short summary, it may encompass a complex interaction between exogenous cannabinoids and the endocannabinoid system, autonomic nervous system, oxidative stress, direct cellular effects on the endothelium, and pro-coagulant effects.^86^ Exposure to THC causes activation of the sympathetic nervous system and inhibition of the parasympathetic nervous system.^87^ These effects include elevated heart rate, serum norepinephrine levels, elevated supine blood pressure, and increases in left ventricular systolic function^87^–^89^ Smoking results in decreasing oxygen delivery to the heart and other vital organs and may be further compromised by increasing carboxyhemoglobin levels.^90^ The impaired myocardial oxygen demand-to-supply ratio following cannabis smoking has been shown to reduce the time to onset of symptoms during exercise in patients with stable angina.^91^

Direct effects of cannabis on blood vessels are complex due to the differing compounds in cannabis and the functional properties of the blood vessels examined.^92^ Studies are inconsistent regarding the effects on vasoconstriction and dilation. Cannabis has been consistently shown to produce vasodilation with resultant orthostatic hypotension,^92^,^93^ but it has also been implicated in vasoconstrictive arteritis mechanisms.^94^,^95^ A large review article suggested that there are three phases in cardiovascular parameters affected by the endocannabinoid system and that different chemical constituents of the cannabis plant have varying effects at different target organs, which may account for the differences.^93^ Transient vasospasm and reduction in cerebral blood flow are well described and may underlie changes in coronary, cerebral, and peripheral arterial systems leading to end organ ischemia.^86^,^96^,^97^ Myocardial blood flow has been shown to correlate inversely with circulating plasma levels of endocannabinoids.^86^,^98^ Cannabis has also been shown to be a potent source of cellular oxidative stress through formation of reactive oxygen species, and this may contribute to endothelial dysfunction and promote regional arterial vasospasm.^86^,^99^

**Figure 7:**
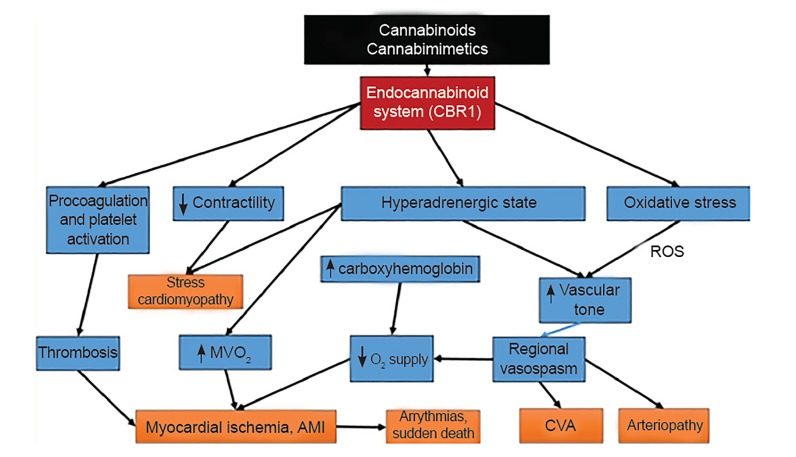
Flow diagram demonstrating pathophysiologic pathways to common major adverse cardiovascular events reported in users of cannabis and related chemicals.^86^ *AMI*, acute myocardial infarction; *CBR1*, cannabinoid receptor 1; *CVA*, cerebrovascular accident; *MVO*_2_ myocardial oxygen consumption (demand); *O_2_*, oxygen; *ROS*, reactive oxygen species.

THC has also recently shown a dose-dependent pro-coagulant effect.^100^ This ex vivo observation has been supported by reports of thrombotic coronary artery occlusion in young individuals without underlying atherosclerosis.^86^ There are also cannabinoid receptors on the surface of platelets and THC has been shown to increase the surface expression of glycoprotein IIb–IIIa and P selectin in a concentration-dependent manner resulting in platelet activation.^86^,^101^ Figure 7 summarizes these effects.

#### Respiratory Effects

Marijuana smoking leads to adverse pulmonary outcomes. The NASEM, CDPHE, and WHO reports state there is substantial evidence of a statistical association between marijuana smoking and worse respiratory symptoms and more frequent chronic bronchitis episodes.^6^,^10^,^75^ These data were based primarily on a systematic review by Tetrault et al. from 14 studies that assessed the association between long-term cannabis smoking respiratory symptoms including chronic cough (OR = 1.7–2.0), increased sputum production (OR = 1.5–1.9), and wheezing (OR = 2.0–3.0).^102^ There is also evidence of a statistical association between the cessation of cannabis smoking and improvements in respiratory symptoms.^10^ Cannabis smoking may also lead to increased rates of pneumonia and upper respiratory infections. On histology, this is associated with a reduction in ciliated cells and increase in mucus secretion from the larger number of mucus-secreting cells.^6^,^30^,^103^

#### Exposures to Children

Reported exposures to children less than age 10 have sharply increased in Colorado following recreational marijuana legalization. A retrospective cohort study of hospital admissions and regional poison control center (RPC) cases between January 1, 2009–December 31, 2015 at a tertiary-care children’s hospital found that the mean rate of marijuana-related visits to the children’s hospital (ages 0–9) increased from 1.2 per 100,000 population in the two years prior to legalization to 2.3 per 100,000 after (P = .02). The median age of exposure was 2.4 years. The majority were exposure to an infused edible product (48%, n = 30); 65% (n = 40) were observed in the ED or UC; 21% (n = 13) were admitted to an inpatient ward; and 15% (n = 9) were admitted to the intensive care unit. Two of these children required respiratory support. The median length of stay for all patients was 11 hours, and the median length of stay for admitted patients was 26 hours.

**Figure 8:**
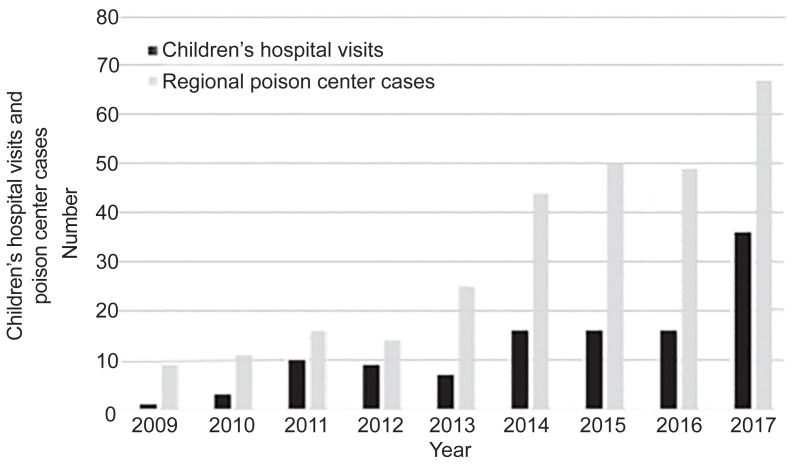
Colorado pediatric marijuana exposures (ages 0–9) to a tertiary-care children’s hospital, and regional poison control center cases by year.^105^

Annual RPC pediatric marijuana cases increased more than five-fold from 2009 (nine cases) to 2015 (47 cases).^104^ Colorado had an average increase in RPC cases of 34% (P < .001) per year while the remainder of the United States had an increase of 19% (P < .001).^104^ In a follow-up study in October 2018, the same author found that despite multiple public health interventions in legislation after 2014 (child-resistant packaging, dose limitations, opaque packaging, limiting marketing campaigns, and banning specific edibles), the incidence of children’s hospital visits and RPC calls has continued to rise in Colorado with an observed doubling of children’s hospital visits in 2017 compared to 2016^105^ (Figure 8). Edibles are sold as cookies, candies, and sodas with advertising that appeals to children.^104^,^106^

#### Cannabis Contaminants, Cannabis Concentrates, and Hash-oil Burns

Varying cultivation techniques and end-product alterations further complicate the understanding of the physiological effects of cannabis. Cannabis plants can be altered to achieve higher growth rates, changes in potency, and increased bud production. These techniques can include use of varying soil types, fertilizers, and pesticides that can result in physiological effects. These changes may also result in exposures to possible fungal agents such as powdery mildew and botrytis; budworm or mite infestations have been reported in the literature. Historically, there have been reports of bacterial contamination with salmonella, enterobacter, streptococcus, and klebsiella, as well as case reports of fungal spore contaminants, including mycotoxin-producing strains of aspergillus.^107^

There are three pathways through which cannabis may be contaminated with heavy metal substances. Firstly, cannabis is able to remove heavy metals from substrate soils and deposit these in its tissues by virtue of its bioaccumulative capacity. Secondly, cross-contamination may occur during processing (eg, during drying). Thirdly, post-processing adulteration may occur, whereby metals may be added to the preparation to increase weight and thereby appreciate its street value. There are case reports of lead and arsenic poisoning from cannabis.^107^ Pesticides are also commonly used in cannabis cultivation. In a report from Washington State, laboratory analysis revealed that 84.6% (N = 26 samples) of legalized cannabis products contained significant quantities of pesticides including insecticides, fungicides, miticides, and herbicides. These comprised a wide array of different substances and encompassed proven carcinogens (carbaryl, diuron, ethoprophos, permethrin, and propargite), endocrine disruptors, as well as a variety of developmental, reproductive, and neurological toxins.^107^,^108^

There are also changes in end-product concentrations through post-processing of the plant. These changes include creation of oils, waxes/shatter, and dabs. Oils are created by removing the hydrophobic components such as THC with a heated butane solvent. THC concentrations may reach up to 55.7%.^109^ Waxes and shatter are concentrated and solidified oil with THC concentration reaching up to 90% THC.^110^,^111^ Dabs are composed of heated wax and are inhaled off of an object such as a nail, which even further concentrates THC content over 90%.^112^

Preparation of these concentrated products has also led to fires and explosion injuries in amateur production attempts in garages, tool sheds, and vacant homes.^113^,^114^ In Colorado 29 patients with butane hash-oil burns were admitted to the University of Colorado Burn Center from 2008–2014. Zero cases presented prior to medical liberalization, 19 during medical liberalization (October 2009–December 2013), and 12 from January–June 2014 at the study’s conclusion. (Two patients had four total visits.) The median total body surface area (TBSA) burn size was 10% (TBSA range 1–90%). Median length of hospital admission was 10 days. Six required intubation for airway protection while 19 required skin grafting.^115^

#### Marijuana Shop Employees Providing Medical Advice

Marijuana shop employees not trained in medicine or pharmacology are giving medical advice that may be harmful to patients. A recent study in Colorado found that employees are giving medical advice 70% of the time to use cannabis for treatment of nausea and vomiting in pregnancy and few dispensaries encouraged discussion with a healthcare provider without prompting.^116^ The author has personally had patients bring in products recommended by dispensary workers with a recommended potency and frequency of use and report being advised to stop their usual medications and use the cannabis product instead. Cannabis dispensaries provide medical advice and offer treatment without medical training even when this may harm the patient.

### Potential Medicinal Uses of Cannabis

There are potential therapeutic intervention targets for cannabinoids. In general, these therapeutic targets require a high ratio of cannabidiol compounds (CBD-cited to decrease or eliminate the psychoactive effects of THC), and are from products that significantly differ from those found in commercial dispensaries. The NASEM report found substantial evidence that cannabis or cannabinoids are effective for the treatment of chronic pain in adults, as an antiemetic for chemotherapy-induced nausea and vomiting, and for improving patient-reported multiple sclerosis spasticity symptoms. They also found moderate evidence that cannabis or cannabinoids are effective for improving short-term sleep outcomes associated with obstructive sleep apnea, fibromyalgia, chronic pain, and multiple sclerosis.^10^ Studies have also demonstrated that cannabinoids may improve cardiovascular outcomes.^92^,^117^

Likely the most significant treatment implication has been in patients with refractory epilepsy, most commonly in patients with Dravet’s syndrome and Lennox-Gestault syndrome, but also in other patients. This has led to the U.S. Food and Drug Administration approving Epidiolex (a high concentration CBD cannabinoid treatment) in June 2018 for the treatment of Dravet’s syndrome and Lennox-Gestault syndrome.^118^–^120^ Despite these potential medicinal uses, current Colorado legal distribution of cannabis products goes through an intermediary budtender before making it to the patient which may not consistently promote therapeutic benefit; there is insufficient training of dispensary staff to serve this purpose.

### Variations in Potency, Bias in Studies, and Conflicting Laws Confuse Consumers and Impair Research

The potential positive health effects of cannabis rest on which of the multiple species and hybrids are studied and their specific chemical composition. One of the difficulties in determining the physiological effects of cannabis is that “marijuana,” or “cannabis,” can refer to multiple species of plants with widely varying chemical compounds and corresponding variable physiological effects. The cannabis genus includes multiple species, most commonly *Cannabis sativa* and *Cannabis indica*, and within those are hybrids specifically developed by growers to achieve a specific effect. For example, the commonly used term, hemp, refers to a variety of *Cannabis sativa* that is fast growing and can be spun into usable fiber for paper, textiles, clothing, biofuel, animal feed, and other industrial uses. Hemp has low concentrations of THC (less than 0.3%) and higher concentrations of CBD.

The differences in composition offer different potential treatment effects. For example, the effect for pain control cited in the NASEM review was primarily found with nabiximols (Sativex), a cannabis extract mouth spray that delivers a dose of 2.7 mg of THC and 2.5 mg of CBD.^121^ For comparison, a typical marijuana cigarette or joint contains 0.5 g of marijuana and THC content ranges from 12–23%; therefore, a typical joint contains 60–115 mg of THC, 20–40 times the medicinal dose. The NASEM cautioned that many of the cannabis products sold in state-regulated markets bear little resemblance to those available for research at the federal level in the U.S.^10^ This is further complicated in that commonly sold cannabis products are often mislabeled for CBD and THC content. One study showed only 17% of dispensary products were accurately labeled.^122^,^123^ Scientific studies, particularly for treatment of pain, have been limited by a substantial bias, and results have varied.^124^,^125^ Some demonstrate improvement in pain^10^ with coinciding decreases in opiate abuse,^10^,^57^,^126^ while others show the opposite.^123^,^125^,^127^,^128^

The conflict between federal and state laws on the medical use of cannabis products, the lack of consistency among state laws, and the availability of artisanal products in dispensaries, with high variability between composition of products, have caused significant confusion for researchers and limited the ability to fully and accurately research the true effects of commonly available dispensary cannabis products.^129^

## LIMITATIONS

This was not a systematic review of the literature but rather a summary of selected research including several large reviews from the NASEM, the WHO, and the CDPHE. There is undoubtedly much literature, some of it conflicting, not cited here. However, as other states and countries wrestle with decriminalization and legalization of cannabis for personal use and sale, it is crucial to report the Colorado experience as a cautionary tale. This review summarizes a large body of research for practicing emergency physicians who are increasingly confronted with questions and patients who use cannabis. Although the author practices in Colorado, the information is likely generalizable. This review clearly reflects the author’s biases, yet its composition was motivated by alarming experience in everyday practice.

Discussions of cannabis’ effects are relevant not only to the healthcare system, but to legal, business, environmental, legislative, and other branches within a public health framework. This article does not address those other facets. Neither have numerous other physiological effects of cannabis been reviewed here. Many of the previous research studies have focused on cannabis with a much lower THC level limiting applicability to cannabis sold at dispensaries today. Finally, the words “marijuana” and “cannabis” were used interchangeably throughout the article. This was done to maintain the wording from the studies cited consistent with their original language. No difference should be implied with the alternating use of these terms.

## CONCLUSION

Cannabis legalization has led to significant health consequences, particularly to EDs and hospitals in Colorado. The most concerning include psychosis, suicide, and other substance abuse. There are deleterious effects on the brain and some of these may not be reversible with abstinence. Other significant health effects include increases in fatal motor vehicle collisions, adverse effects on cardiovascular and pulmonary systems, inadvertent pediatric exposures, cannabis contaminants exposing users to infectious agents, heavy metals, and pesticides, and hash-oil burn injuries due to preparation of concentrates. Finally, cannabis dispensary workers not trained in medicine are giving medical advice that could be harmful to patients.

Cannabis research may offer opportunities for novel treatment of seizures, spasticity from multiple sclerosis, nausea and vomiting from chemotherapy, chronic pain, improvements in cardiovascular outcomes, and sleep disorders. However, progress has been difficult due to absent standardization of the chemical composition of cannabis products and limitations on research secondary to federal classification of cannabis. Given these factors and the Colorado experience, other states should carefully evaluate whether and how to decriminalize or legalize non-medical cannabis use.
